# Of Those We Have Lost and Those Who Have Saved So Many Others

**DOI:** 10.3201/eid2807.AC2807

**Published:** 2022-07

**Authors:** Terence Chorba

**Affiliations:** Centers for Disease Control and Prevention, Atlanta, Georgia, USA

**Keywords:** art science connection, emerging infectious diseases, art and medicine, poetry, about the cover, modernism, of those we have lost and those who have saved so many others, poem of February 9, 1915, Guillaume Apollinaire, pandemics, coronavirus, severe acute respiratory syndrome coronavirus 2, SARS-CoV-2, influenza, human, influenza A virus, viruses, coronavirus disease, COVID-19, respiratory infections, H5N1 subtype, zoonoses

**Figure:**
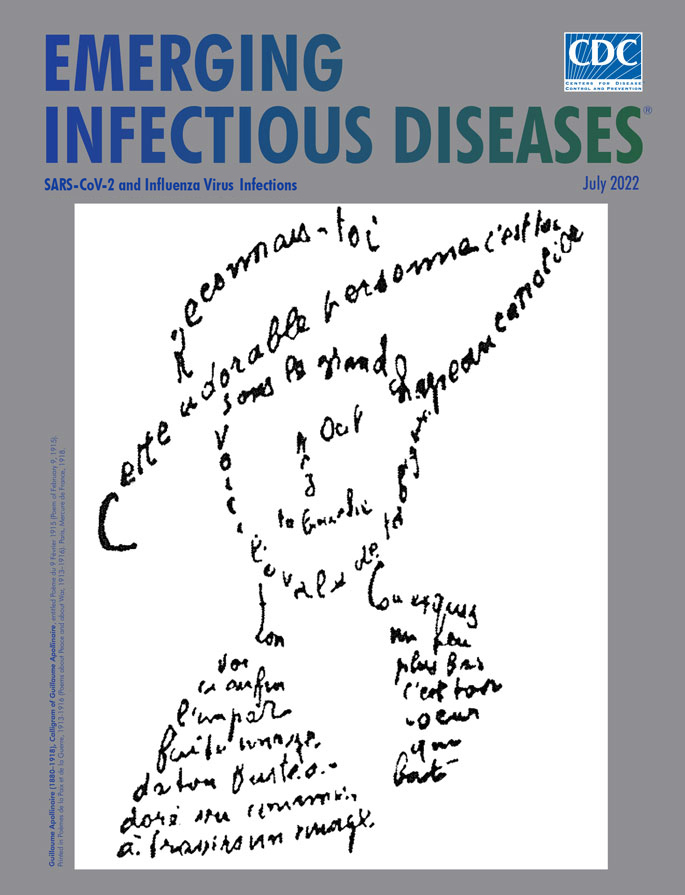
**Guillaume Apollinaire (1880‒1918), Calligram of Guillaume Apollinaire, entitled Poème du 9 Février 1915 (Poem of February 9, 1915).** Printed in Calligrammes:Poèmes de la Paix et de la Guerre 1913–1916 (Calligrams: Poems of Peace and War 1913–1916). Paris, Mercure de France, 1918.

Modernism is a term ascribed to styles and transformative movements in multiple cultural spheres—philosophy, music, art, architecture, and literature. In its essence, modernism has at its core experimentation, as a term usually applied to efforts and creations of the late 19th or early 20th century, but sometimes later, characterized by intentional departures from traditional forms. 

There are many well-known examples of modernist efforts in their respective spheres and periods. In biology, Charles Darwin questioned the concept of human uniqueness with the theory of evolution. In literature, the term modernist has been applied to European and American writers who created substantive departures from tradition, as was seen in the works of Fyodor Dostoyevsky, Gustave Flaubert, James Joyce, and William Carlos Williams. In music, modernism is a term ascribed to the period 1890–1930, and postmodernism is a term sometimes accorded to phenomena with modernist qualities but occurring after 1930; however, some critics use modernism to describe a movement of rebellion that continues, dependent on the musician’s attitude rather than the musician’s moment in time. Certainly, Ella Fitzgerald, Miles Davis, Bob Dylan, John Lennon, Charles Mingus, the Rolling Stones, and Neil Young created musical forms that featured modernist iconoclasm, stepping well beyond the early 20th century. In art, modernism is used as a broader categorization of several novel stylistic departures including realism, postimpressionism, fauvism, cubism, dadaism, surrealism, abstract expressionism, and minimalism, each with elements of deliberate experimentation and innovation.

Guillaume Apollinaire (1880‒1918) was a renowned Belarus-born French poet and critic of modernist art; he created the terms “cubism” in 1911 and “surrealism” in 1917. Apollinaire was also a pioneer in his period for his collection of shape poems called calligrams, published in 1918 in *Calligrammes: Poemès de la paix et de la guerre 1913–1916 (Calligrams: Poems of Peace and War 1913–1916*). Shape poems, also known as concrete poems, are poetic works that are shaped like their subjects or topics. Calligrams are a subset of this genre in which the text is thematically arranged such that the visual image of what is written or typed closely reflects what the words themselves express. The image on this month’s cover is one such cal­ligram that described how Apollinaire was enamored of his beloved, using a female visual image and illus­trating the eye (*oeil*), nose (*nez*), mouth (*bouche*), and neck (*cou*) using those very words (in French) in the visual representation of those parts of the image: “*Reconnais-toi. Cette adorable personne c’est toi. Sous le grand chapeau canotier. Nez. Oeil. Ta bouche. Voici l’ovale de ta figure. Ton cou exquis. Voici enfin l’imparfaite image de ton buste adoré vu comme à travers un nuage. Un peu plus bas c’est ton coeur qui bat.”* [“You recognize that this lovely person, that’s you, under a wide boater’s hat….Nose…Eye…Your mouth. Here, the oval of your face…your exquisite neck. And finally, here is the flawed picture of your beloved bust seen as through a mist. And down a little farther is your heart that is beating.”]

Although Apollinaire’s calligrams are thought of as a departure from other formulaic categories of poetry (e.g., haiku or the Shakespearean sonnet) elements of such calligraphic experimentation and innovation have been found in older cultures. As an Alexandrian poet of the fourth century bce, Simmias of Rhodes has been renowned for three shape poems written (in Greek) in the shapes of a pair of wings (πτέρυγες), an egg (ώόν), and a hatchet (πέλεκυς). During the Middle Ages, micrography in the form of Hebrew and Muslim shape poems was developed, sometimes serving as a workaround for religious restrictions on graven images, to remain devout in observation of Jewish or Islamic law. A more recent historical example is “The Mouse’s Tale,” a visually shaped poem in Lewis Carroll’s *Alice in Wonderland*, in which the mouse begins to tell Alice his story by saying, “Mine is a long and a sad tale!", leading Alice to think that the mouse is referring to its tail (Figure). For all these authors, the trick of the shape poem, especially the calligram, was to create a poem that depends both on form and words for meaning, with primacy given over to the visual effect, as a synthesis of visual art and lyrics. 

**Figure:**
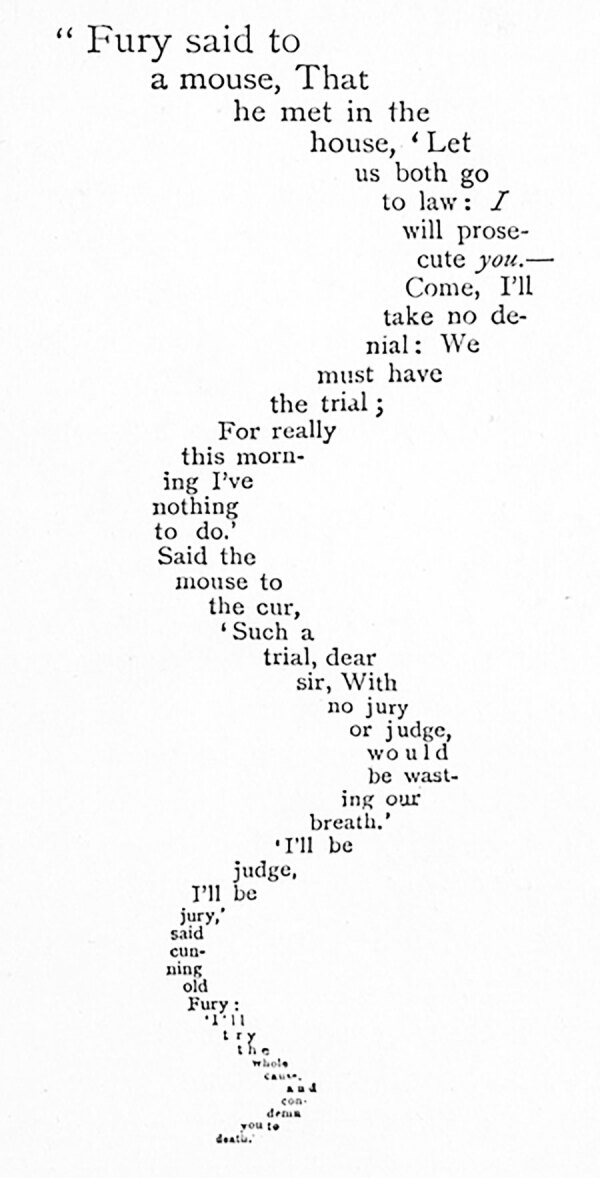
Printed version of “The Mouse’s Tale” (*3*). p. 36 in the 1865 edition of *Alice's Adventures in Wonderland.* Source: Wikipedia.

On November 9, 1918, two days before the armistice of the First World War, Apollinaire died at age 38. He was but another casualty in the second wave of the great influenza A (H1N1) pandemic that had begun in the winter months of early 1918, and his death was characteristic of most deaths documented in that pandemic, occurring among those in their childbearing years. Persons 40–65 years of age tended to have relative immunity to this pathogen, the result of virus exposure decades earlier. That pandemic helped to end the four years of conflict in Europe in which more died of infectious disease than of combat wounds themselves. Two other great poets, both renowned in English-speaking circles, were lost in that war to infectious disease: John McCrae, who died of meningitis (*In Flanders Fields*— *“*In Flanders fields the poppies blow between the crosses, row on row”), and Rupert Brooke, who died of septicemia resulting from an infected mosquito bite (*The Soldier* “If I should die, think only this of me: That there’s some corner of a foreign field that is forever England”). Going beyond the war into 1919, about 500 million persons became infected with the circulating influenza virus worldwide, comparable to estimates of the number of persons who have become infected to date with SARS-CoV-2, the causative agent of COVID-19. 

Pandemics and wars leave vacuums in all sectors, and the arts are no exception. In the current COVID-19 pandemic, among celebrated musicians alone, we have lost John Prine, Trini Lopez, Charley Pride, Mamu Dibango, Antoine Hodge, and many others. As the death toll from COVID-19 has exceeded 1 million in the United States and 6,250,000 worldwide, myriad preventive and therapeutic measures that have evolved over the past century, including vaccines, antivirals, antibiotics, and monoclonal antibodies, have kept this grim tally from growing even worse. During the ongoing SARS-CoV-2 pandemic, modern healthcare resources have saved millions of lives, including those of many creative souls—musicians, writers, poets, illustrators, sculptors, painters, and graphic artists—in contrast to conditions during the influenza A pandemic at the end of the First World War. The work of many heroes, including research scientists, public health responders, administrators, healthcare providers, support personnel, and advocates, should be acknowledged and celebrated.
